# Associations between health-related quality of life, physical function and fear of falling in older fallers receiving home care

**DOI:** 10.1186/s12877-018-0945-6

**Published:** 2018-10-22

**Authors:** Maria Bjerk, Therese Brovold, Dawn A. Skelton, Astrid Bergland

**Affiliations:** 1 Department of Physiotherapy, OsloMet – Oslo Metropolitan University, PO Box 4 St. Olavs plass, 0130 Oslo, Norway; 2 School of Health and Life Sciences, Glasgow Caledonian University, Glasgow, UK

**Keywords:** Health-related quality of life, Falls, Falls-efficacy, Fear of falling, Home care

## Abstract

**Background:**

Falls and injuries in older adults have significant consequences and costs, both personal and to society. Although having a high incidence of falls, high prevalence of fear of falling and a lower quality of life, older adults receiving home care are underrepresented in research on older fallers. The objective of this study is to determine the associations between health-related quality of life (HRQOL), fear of falling and physical function in older fallers receiving home care.

**Methods:**

This study employed cross-sectional data from baseline measurements of a randomised controlled trial. 155 participants, aged 67+, with at least one fall in the previous year, from six Norwegian municipalities were included. Data on HRQOL (SF-36), physical function and fear of falling (FES-I) were collected in addition to demographical and other relevant background information. A multivariate regression model was  applied.

**Results:**

A higher score on FES-I, denoting increased fear of falling, was significantly associated with a lower score on almost all subscales of SF-36, denoting reduced HRQOL. Higher age was significantly associated with higher scores on physical function, general health, mental health and the mental component summary. This analysis adjusted for sex, education, living alone, being at risk of or malnourished, physical function like balance and walking speed, cognition and number of falls.

**Conclusion:**

Fear of falling is important for HRQOL in older fallers receiving home care. This association is independent of physical measures. Better physical function is significantly associated with higher physical HRQOL. Future research should address interventions that reduce fear of falling and increase HRQOL in this vulnerable population.

**Trial registration:**

ClinicalTrials.gov. NCT02374307. First registration, 16 February 2015. First enrolment of participants, February 2016.

## Background

The increasing number of older adults living longer poses new challenges to health, long-term care and the welfare system [1]. The rising costs of falls and associated injuries are of global concern [2], estimated at 1.5% of health care costs in European countries, both directly from the fall-related injuries and indirectly through loss of mobility, confidence and functional independence [3]. Costs for long-term care are expected to increase substantially in the future. These expenses can be greatly reduced if the older adults are in good health and are able to remain at home [1]. Home care services are important in maintaining independence, contributing to functional health status and improving the quality of life (QOL) among older adults [4].

Home care is here defined as services provided by health professionals to people in their own homes and can cover a wide range of activities, from care related to individual needs to preventative assessments and actions [4]. The population of home care recipients constitutes a transitional group between independent community living older people, and people living in residential care facilities, and their health-related quality of life (HRQOL) and other health outcomes might be different from those [5]. Even though home care could be an important contributor meeting the challenges of an increasing older population, surprisingly few clinical studies have been carried out including this group of older fallers [6, 7]. Falls and disability are strong predictors of institutionalisation. By targeting home care recipients who have experienced falls, the frequency of nursing home admissions could be reduced [8].

In Norway, the municipalities are responsible for providing home care for older adults, and recent governmental guidelines have put more focus on these services to enable older adults to remain at home as long as possible [9]. Home care comprises services like home nursing, practical assistance with daily activities and safety alarm. Home nursing and assistance with personal care are free of charge, while practical assistance and safety alarm services have deductibles. In 2016, 12% of the Norwegian population in the age group 67–79 years received home care services. In the age group 80–90 years, the share was 50%, and 90% for those 90 years or older [10]. Across Europe, health services at home are becoming increasingly important [1]. WHO guidelines point out a change in focus of clinical care for older adults globally, where community and home-based care are emphasised [11].

The literature on falls in the general population of older adults is extensive. Home care receivers and other groups of frailer older adults are still underrepresented in this literature [12]. Older adults receiving home care services have a high incidence of falls, with 10% experiencing multiple falls during the previous 90 days [13]. The level of services provided correlates with the incidence of falls [14]. This group of older adults also report a high prevalence of fear of falling and activity restrictions associated with this fear [15]. In the general population of older adults, fear of falling and its consequences have been identified as important factors influencing HRQOL [16–18]. This relationship has not been established in the population of older home care receivers. It can be expected that receiving care and support could have an impact on the level of fear of falling and on HRQOL. Thus, this group of frailer older adults might be different than the general group of older adults when looking at the relationship between HRQOL and fear of falling.

The general population of fallers scores significantly lower on HRQOL, in particular on the physical component [19]. HRQOL has been shown to be associated with measures of mobility, balance and pain [20]. In the population of older adults receiving home care, studies looking specifically at HRQOL and further associations to physical function is lacking. However, studies exploring a broader concept, QOL, show that it is lower in this population compared to older adults in the same age group [21]. Among home care recipients, higher QOL has been associated with higher age, not living alone, a lower number of complaints like pain or impaired mobility, and managing to be alone at home [22]. Despite finding an association between mobility and QOL, HRQOL was not explored and different factors of physical activity as balance, walking speed or muscle strength were not included.

The complexity of the health challenges in the group of older fallers receiving home care makes it challenging for those delivering primary health care, both to ensure HRQOL for the client and at the same time keeping the costs reasonable [23]. There is a knowledge gap in clinical research on HRQOL and falls including older adults receiving home care [5, 24]. In recent guidelines, both locally in Norway, but also internationally, policy makers are increasingly focusing on the challenges of organising effective and high-quality health care services to meet the needs of the population of older home care recipients [9, 11]. In order to develop services and interventions, thorough information on the health status of this population is needed. The objective of this study is therefore to determine relationships between HRQOL and fear of falling as well as physical function in older fallers receiving home care services.

## Method

### Study design

The analysis employs cross-sectional data from baseline measurements of a randomised controlled trial conducted in 2016–17 [24]. The trial was registered at ClinicalTrials.gov in February 2015, NCT02374307. First enrolment of participants was in February 2016. The STROBE guidelines are followed to report on the design, analysis and presentation of data [25].

### Setting and participants

**Fig. 1:**
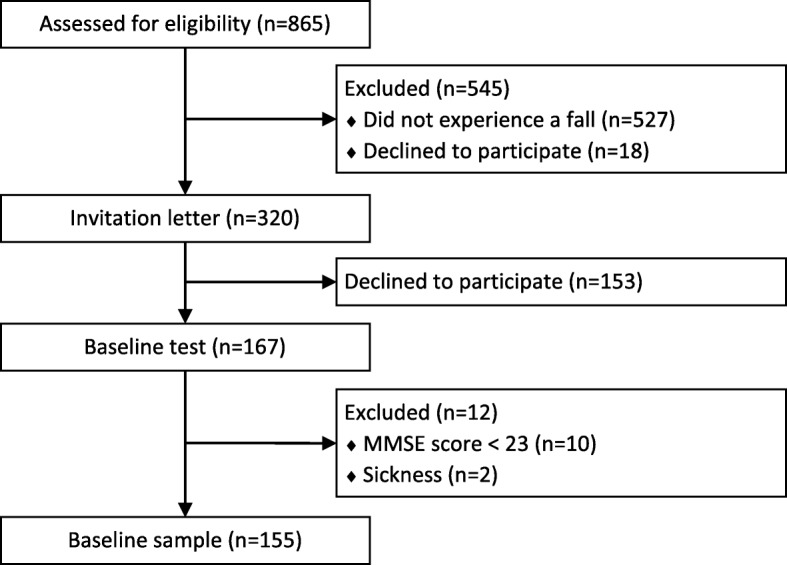
Flow of participants

Participants were recruited in six municipalities in Norway. Recruitment was based on registration lists of older adults receiving home care from primary health care services. The recruitment plan is described elsewhere [24]. The flow of participants at enrolment in the project is illustrated in Fig. 1. Eight hundred sixty five adults receiving home care were initially assessed for eligibility, 320 received an invitation letter and 167 were baseline tested. Data from 155 participants were included in the final sample analysed in this study.

The study was approved by the Regional Committee for Medical Research Ethics in South Norway (Ref. 2014/2051). Participants provided written, informed, consent.

#### Inclusion criteria

Age 67+, receiving home care from the primary health care services, having experienced at least one fall in the last 12 months, able to walk with or without a walking aid and understand Norwegian.

#### Exclusion criteria

Medical contraindications to exercise, life expectancy less than 1 year, a score below 23 on the Mini Mental State Examination (MMSE) and participating in other falls prevention programmes.

### Outcome measures

The outcome measures for this study were selected based on both theoretical and practical reasons [26, 27]. All assessments employed have established reliability and validity, as recommended by the CONSORT guidelines [28]. In addition to improving measurement quality and outcomes, it enables direct comparisons with other studies investigating HRQOL and can possibly contribute to future meta-analyses. The measurements were conducted by physiotherapists in the participants’ home in one session, so considerations had to be made both concerning equipment and fatigue of the participants.

***Health-related quality of life*** was assessed using the Short Form 36 Health Survey, version 2 (SF-36). This questionnaire is generic, validated and translated into Norwegian [29]. The 36 items in SF-36 are grouped into eight subscales: physical functioning (PF), role limitations due to physical problems (RP) and due to emotional problems (RE), bodily pain (BP), general health (GH), vitality (VT), social functioning (SF) and mental health (MH). Based on the scores of these eight scales, a physical component summary (PCS) and a mental component summary (MCS) is calculated. The sum scores range from 0 to 100 (worst-best).

***Fear of falling*** was measured using the Norwegian version of the Falls Efficacy Scale International (FES-I) [30]. In FES-I fear of falling is operationalised as the level of concern about falling when carrying out a range of 16 different physical activities [31]. It has a four-point scale ranging from 1 (not concerned) to 4 (very concerned). A sum score between 16 and 64 is achievable, where 16–19 indicates low concern, 20–27 moderate concern and 28–64 high concern [31].

***Physical function*** was assessed by measurements on balance, gait speed, muscle strength and instrumental activities of daily living (IADL).

The Berg Balance Scale (BBS) assesses balance. The Norwegian version has been shown to have an excellent inter-rater reliability and high internal consistency in the geriatric population [32]. BBS measures performance on a 5-level scale from 0 (cannot perform) to 4 (normal performance) on 14 different tasks. The sum score of the 14 items ranges from 0 to 56, where a score below 45 indicates that the individual has a higher risk of falling.

Gait speed was assessed based on the time required to walk 4 meters, using any usual walking aid, and expressed in meters per second [33].

Muscle strength was measured by using the functional proxy measure of 30 seconds sit-to-stand (STS) test, where the number of rises from a chair within 30 seconds is recorded [34].

IADL was measured using the Norwegian version of the Lawton IADL scale [35]. It assesses a person’s self-reported ability to perform complex activities of daily living. There are eight areas of function that are assessed, and the summary scores ranges from 0 (low function) to 8 (high function).

***Demographic and background variables*** were age, sex, living alone, education (primary and lower secondary school/upper secondary school/university 1–4 years/university more than 4 years), medical history including medications, nutritional status measured by Mini Nutritional Assessment (MNA) [36], walking aid use, type of home care (home help/home nursing/safety alarm service) and history of falls.

### Data analysis

Statistical analyses were conducted using STATA/SE 14. Descriptive characteristics of the study population are reported. Percentages are used to describe categorical data, and mean and standard deviation (SD) are calculated for continuous data. Skewness was examined by comparing mean and median values. Differences between males and females were inspected by t-tests and χ^2^ tests. Coefficients with *p*- values ≤0.05 were considered statistically significant.

Pearson correlations coefficients display the association between the subscales of SF-36 and measures of physical function and fear of falling. The strength of correlations was interpreted according to Cohen, where 0.10 to 0.29 is weak, 0.3 to 0.49 is moderate and 0.5 to 1.0 is strong [37].

Explanatory variables for the multivariate regression of the scales of SF-36 were chosen from the available set of variables displayed in Table 1. The regressions adjust for the background variables age, sex, education, living alone, risk of or being malnourished, falls ≥3 during the previous 12 months and the number of different medications. The minimum values from the inclusion criteria were subtracted from age (67) and MMSE (23) to increase interpretability of the coefficients. A dummy variable was created for more than two falls in the last 12 months. Most participants had one or two falls, while some had a large number of falls. Additionally, the regression included as independent variables 4-m walk test (4MWT), BBS, IADL, FES-I and MMSE. STS was highly correlated (> 0.6) with both BBS and 4MWT and this variable was therefore excluded from the regressions. The impact of the variables health care services and walking aid were negligible, and those were also excluded. Four records containing missing observation of medications and 4MWT had to be dropped.

**Table 1:** Characteristics of the study population. Means, standard deviations (SD) and percentages

	Total (*N* = 155)	Female (*N* = 123)	Male (*N* = 32)
Characteristics			
Age, mean (SD)	82.7 (6.7)	83.0 (6.7)	81.3 (6.7)
Living alone, %	84.5	87.0	75.0
Higher education (> 12 years), %	36.1	35.0	40.6
No. of medications weekly, mean (SD)	5.3 (3.4)	5.1 (3.4)	6.0 (3.6)
Primary health care services			
Practical assistance, %	69.7	68.3	75.0
Nursing, %	30.3	27.6	40.6
Safety alarm service, %	75.5	79.7	59.4
Walking aid %	73.5	74.0	71.9
Falls the last 12 months			
No., mean (SD)	2.7 (3.7)	2.1 (2.5)	4.9 (6.0)
Location:			
Indoor, %	47.4	49.6	38.7
Outdoor, %	18.8	19.5	16.1
Both, %	33.8	30.9	45.2
Injuries from falls:			
Minor injuries %	45.5	45.5	45.2
Serious injuries, hospitalisation %	35.1	37.4	25.8
Mini-Mental State Examination			
MMSE, mean (SD)	27.4 (2.2)	27.5 (2.2)	27.2 (2.2)
Falls Efficacy			
FES-I, mean (SD)	30.7 (9.8)	31.0 (9.9)	29.4 (9.5)
Physical function			
IADL, Lawton and Brody. > 6, %	56.1	56.1	56.3
Sit to stand, mean (SD)	5.1 (4.1)	5.1 (4.2)	4.8 (3.7)
4-m walk test m/s, mean (SD)	0.62 (0.21)	0.62 (0.22)	0.61 (0.18)
Berg Balance Scale, mean (SD)	39.1 (11.3)	39.6 (11.4)	37.2 (10.8)
Mini Nutritional Assessment			
Risk of or malnourished %	24.4	27.6	12.5
Health-related quality of life			
SF-36 scores, mean (SD)			
Physical component summary	38.3 (9.0)	38.0 (9.2)	39.4 (8.4)
Mental component summary	49.4 (10.3)	49.0 (10.6)	50.9 (9.1)
Physical function	44.6 (23.1)	44.5 (23.0)	45.2 (23.8)
Role physical	51.7 (29.7)	50.9 (30.1)	54.9 (28.3)
Body pain	53.8 (32.2)	51.8 (32.4)	61.4 (30.7)
General health	57.6 (23.3)	57.6 (23.5)	57.6 (22.7)
Vitality	38.3 (21.5)	36.7 (28.8)	44.2 (19.1)
Social function	66.9 (31.2)	66.1 (31.3)	69.9 (30.8)
Role emotional	75.8 (28.5)	75.6 (28.1)	76.6 (30.6)
Mental health	72.1 (17.4)	71.1 (17.8)	75.6 (15.6)

Floor- and ceiling effects were considered when more than 20% of the participants achieved the lowest or highest possible score. For RE, 48.4% reached the top score of 100. In this case, a logistic regression was fitted.

## Results

### Participants

Table 1 presents the characteristics of the total sample and separately for females and males. The study included 123 females and 32 males. The only statistical significant difference between sexes was found on the number of falls and if a safety alarm service was provided. Men had a significant higher rate of falls, 4.9, compared to women, 2.1 (*p* < 0.001). Women received a safety alarm more often, 79.7%, than men, 59.4% (*p* = 0.017). Mean (SD) age is 82.7 (6.7). HRQOL, measured by SF-36, shows a better summary score on the mental components (49.4, SD 10.3) than on the physical components (38.3, SD 9.0).

### Correlation coefficients

In Table 2, the correlation coefficients between subscales of SF-36 and different measures of physical function and fear of falling are presented. All measures of physical function are highly correlated with the subscale PF (*p* < 0.01). FES-I is moderately negatively correlated with all subscales of SF-36, except from BP and SF, where there is a weaker negative correlation.

**Table 2:** Correlation between HRQOL (SF-36) and different measures on physical function and falls efficacy

SF-36 subscales	Sit to stand	4 Meter Walk Test	Berg Balance Scale	Instrumental ADL	Falls Efficacy Scale - I
Physical Function	0.515^***^	0.537^***^	0.585^***^	0.439^***^	−0.425^***^
Role Physical	0.352^***^	0.275^***^	0.287^***^	0.250^**^	−0.388^***^
Bodily Pain	0.113	0.146	−0.013	− 0.036	− 0.221^**^
General Health	0.270^***^	0.168^*^	0.175^*^	0.120	− 0.367^***^
Vitality	0.193^*^	0.175^*^	0.116	0.110	−0.327^***^
Social Function	0.267^***^	0.123	0.216^**^	0.210^**^	−0.262^***^
Role Emotional	0.289^***^	0.120	0.201^*^	0.134	−0.355^***^
Mental Health	0.225^**^	0.100	0.082	0.056	−0.362^***^

* *p* < 0.05 **p < 0.01 ***p < 0.001

### Multivariate regressions

Table 3 presents results of multivariate regressions of scales of SF-36 on background variables and measures of physical function and fear of falling. Having a lower score on FES-I is significantly associated with achieving a higher score on all subscales of SF-36 except from BP and SF. Scoring 10 points lower on FES-I, is expected to increase the scores of SF-36 between 0.9 (RE) to 7.3 (RP). The subscale PF is significantly associated with higher scores on the physical measures 4MWT (*p* ≤ 0.05), BBS (*p* ≤ 0.001) and IADL (*p* ≤ 0.01). Higher age is significantly associated with better scores on MCS (*p* ≤ 0.05), PF (*p* ≤ 0.05), GH (*p* ≤ 0.01) and MH (*p* ≤ 0.01). Taking fewer medications is significantly associated with a higher score on PCS (p ≤ 0.001) and GH (*p* ≤ 0.001). Finally, a higher MMSE score is significantly associated with a higher score on MH (*p* ≤ 0.05).

**Table 3:** Regression of SF-36 on measures on demographics, physical measures, cognition and falls efficacy

	Physical Comp. Summary	Mental Comp. Summary	Physical Function	Role Physical	Bodily Pain	General Health	Vitality	Social Function	Role Emotional	Mental Health
Age (years ≥67)	0.19	0.31^*^	0.49^*^	0.58	0.80	0.74^**^	0.04	0.70	0.02	0.64^**^
	(0.10)	(0.13)	(0.23)	(0.37)	(0.42)	(0.28)	(0.28)	(0.42)	(0.03)	(0.22)
Falls ≥3 last 12 months	2.48	−4.23^*^	4.57	1.43	3.90	1.06	−4.37	−9.02	−0.27	−4.64
	(1.56)	(1.99)	(3.51)	(5.49)	(6.36)	(4.17)	(4.22)	(6.28)	(0.46)	(3.27)
No. medications weekly	−0.72^***^	0.16	−0.74	−1.02	−1.10	−2.65^***^	− 0.58	0.43	0.06	−0.33
	(0.19)	(0.24)	(0.42)	(0.66)	(0.77)	(0.50)	(0.51)	(0.76)	(0.06)	(0.39)
4 Meter Walk Test, m/s	8.28^*^	− 1.03	21.12^*^	15.30	23.88	−0.24	16.53	−1.26	0.12	4.37
	(3.84)	(4.88)	(8.61)	(13.47)	(15.62)	(10.24)	(10.35)	(15.43)	(1.18)	(8.03)
Berg Balance Scale	0.14	0.00	0.80^***^	0.31	−0.12	0.18	−0.23	0.33	0.03	−0.00
	(0.08)	(0.10)	(0.18)	(0.27)	(0.32)	(0.21)	(0.21)	(0.31)	(0.02)	(0.16)
Instrumental Activities of Daily Living	0.50	−0.11	3.16^**^	2.01	−1.48	−0.96	0.16	2.36	0.02	− 0.85
	(0.51)	(0.65)	(1.15)	(1.80)	(2.08)	(1.37)	(1.38)	(2.06)	(0.15)	(1.07)
Falls Efficacy Scale – International	−0.18^*^	−0.30^**^	−0.37^*^	− 0.73^**^	−0.55	− 0.55^**^	−0.63^**^	− 0.46	−0.09 ^***^	− 0.52^***^
	(0.07)	(0.09)	(0.16)	(0.25)	(0.29)	(0.19)	(0.19)	(0.29)	(0.02)	(0.15)
Mini-Mental State Examination (score ≥ 23)	−0.26	0.45	−0.11	− 0.38	−1.42	0.70	0.78	0.44	−0.03	1.25^*^
	(0.29)	(0.37)	(0.66)	(1.03)	(1.19)	(0.78)	(0.79)	(1.18)	(0.09)	(0.61)
R^2^ adj.	0.32	0.15	0.47	0.21	0.08	0.27	0.10	0.07		0.20

Additionally adjusted for sex, education, living alone, risk of or being malnourished. Ordinary least squares (OLS) regressions, except on role emotional, where a logistic regression is fitted. Unstandardised regression coefficients, standard error (SE) in parentheses. Model fit reported by R^2^-adjusted. *N* = 151. * *p* < 0.05 **p < 0.01 ***p < 0.00

## Discussion

The objective of this study was to determine the relationship between HRQOL, fear of falling and physical function in older fallers receiving home care. The results show that a higher level of HRQOL, measured by SF-36, is substantially associated with lower fear of falling, measured by FES-I. The associations are independent of physical measures like BBS and 4MWT, number of falls, cognition and key background characteristics. All associations are statistically significant in almost all scales of SF-36, except BP and SF. On physical function, the results show that a higher score on the subscale PF is significantly associated with better gait speed (4MWT), improved balance (BBS) and better ability in IADL.

The present study extends the results of two previous studies on the association between HRQOL and fear of falling. In a Canadian study of older community-dwelling women, quality-adjusted life years were calculated from the EQ-5D scale and compared to falls-related self-efficacy [17]. This study accounted for similar control variables and found comparable results on their measure of HRQOL. However, the women included did not necessarily experience a fall and it was uncertain whether the results could be generalised to older adults with a lower level of function. Another study from Taiwan reported on the association between HRQOL, measured by summary scores of SF-36, and fear of falling [16]. This larger survey included both fallers and non-fallers and adjusted for some background characteristics. Fear of falling was measured simply by asking a yes/no question. Unlike the study by Davis et al. [17] and this present study, the association was not independent of physical or cognitive function. Here, the results show that fear of falling, measured by a validated and reliable instrument, is independently associated with almost all scales of SF-36 and thus confirms that it is an important predictor of HRQOL in this group of older fallers with poor function.

All measures of physical function and IADL were significantly associated with the physical subscale of HRQOL. A higher PF was significantly associated with higher scores on the physical measures 4MWT, BBS and IADL. Similar results have been shown in previous studies where lower HRQOL was associated with difficulties with basic and instrumental activities of daily living [38, 39], low maximal gait speed [40] and reduced physical fitness [41]. The present study did not show any significant associations on other subscales, but the sample size could have been too low to detect other associations.

Research on older adults often excludes those who are frailer [7]. In previous studies, participants were younger than here, where the mean age is 82.7. Research on older fallers has been carried out, but those who receive home care are underrepresented. Risk factors and incidence of falls in this population have received most attention [13, 14]. Associations between HRQOL and potentially influential factors have not been analysed in this group. QOL among older adults receiving home care has been explored in Sweden [21]. In this study, the extent of help with IADL influenced QOL negatively, while it was positively influenced by the density of the social network. Measures of physical and cognitive function were not included in the Swedish study which was based on a postal questionnaire.

Compared to normative values from a Norwegian sample of adults, aged 70 to 80 years, the sample in the present study has lower values in all subscales of SF-36 [42]. This might be due to better function of older people in the general population, not necessarily requiring home care. Similar findings were demonstrated in a Swedish study, where elderly receiving home care had very low QOL compared to older adults in the same age group [21].

Interestingly, higher age was associated with better scores within the scales MCS, PF, GH and MH. This might be due to what has been described in literature on HRQOL as response shift [43]. It refers to a change in the meaning of one’s self-evaluation of HRQOL resulting from changes in internal standards, values and conceptualisation. The oldest of the participants might have lower expectations of their everyday life, what they can manage and their health status, while the younger participants might on average have higher expectations. An earlier Swedish study on QOL of older people living at home found comparable results. High QOL was related to higher age, lower number of complaints and managing to live alone at home [22].

This study has several limitations. First, the sample comprised participants recruited to a controlled trial to potentially perform a falls prevention programme. The participants might be fitter and more motivated for physical activity than the general population of older adults receiving home care. To improve generalisability, recruitment was outreaching, calling from lists of people receiving home care. Half of those who were eligible to participate and sent an invitation letter were also included in the study. This could make self-selection of more active participants less likely. Secondly, the sample was recruited from only six municipalities which are not necessarily representative for Norway in general. However, the six municipalities included both cities and rural areas. Thirdly, performing subgroup analyses on sex is difficult as a low percentage of the sample were males. The descriptive statistics show, however, that males and females in this sample are not significantly different, except for number of falls and if a safety alarm is provided. A further limitation is that the study is cross-sectional and definitive causal relations cannot be established. Finally, some of the measures like the number of falls are self-reported.

This study contributes new knowledge on the level of HRQOL, physical function and fear of falling in addition to the relationship between these factors in a group of older fallers receiving home care. This population is understudied and more information is needed to be able to improve care and other public services for this group. The results from this study can be of importance for clinicians and health managers for developing interventions and organising clinical services in primary health care. Since this group of older fallers is relatively large in Norway and other developed countries, the information can also be useful for policy makers to set priorities and allocate resources. Future research on interventions on how to modify HRQOL and fear of falling within this group is needed.

## Conclusions

Higher HRQOL is substantially associated with a lower level of fear of falling in older fallers receiving home care. This association is independent of physical measures, number of falls, cognition and key background characteristics such as age, sex and education. Better physical function is significantly associated with higher physical HRQOL, independent of the same background characteristics and fear of falling.

## Abbreviations

4MWT: 4 Meter Walk Test
BBS: Berg Balance Scale
BP: Bodily Pain
EQ-5D: EuroQol - five dimensions scale
FES-I: Falls Efficacy Scale - International
GH: General Health
HRQOL: Health-related Quality of Life
IADL: Instrumental Activities of Daily Living
MCS: Mental Component Summary
MH: Mental Health
MMSE: Mini Mental State Examination
PCS: Physical Component Summary
PF: Physical Function
QOL: Quality of Life
RE: Role Emotional
RP: Role Physical
SD: Standard Deviation
SF: Social Function
SF-36: Short From 36 Health Survey
STS: Sit to Stand
VT: Vitality
